# A review of psychocutaneous disorders from a psychotherapeutic perspective—Toolkit for the dermatologist

**DOI:** 10.1002/ski2.211

**Published:** 2023-01-09

**Authors:** Mary Zagami, Edward Klepper, Eric Wienecke, Maria Andrzejewski, Ahmed Sikder, Ali Ahmed, Howard Robinson

**Affiliations:** ^1^ Robinson & Max Dermatology PA Lutherville‐ Timonium Maryland USA

## Abstract

**Introduction:**

The study of psychocutaneous disorders requires a multidisciplinary approach. It is of paramount importance that dermatologists understand the psychiatric premise for these disorders. Mental health professionals can also benefit from a better understanding of the dermatologic manifestations of psychiatric disorders they may encounter in their practice.

**Aim:**

The aim of this study is to elevate the level of understanding regarding psychotherapeutic treatment of psychocutaneous disorders.

**Methods:**

We performed a literature review using the major databases. Four researchers reviewed English, full text, peer‐reviewed articles that were published after 2000 using our specific search terms and inclusion/exclusion criteria.

**Results:**

The majority of psychocutaneous disorders seem to be clustered among three DSM‐5 disorders: depressive disorders, anxiety disorders, and obsessive‐compulsive related disorders.

**Conclusions:**

Better recognition of the underlying psychiatric comorbidities may lead to improved patient outcomes.

## Graphical abstract

Psychocutaneous disorders require a multidisciplinary approach. Better recognition of the underlying psychiatric comorbidities may lead to improved patient outcomes. We hope this review will serve as a bridge between dermatology and psychiatry.

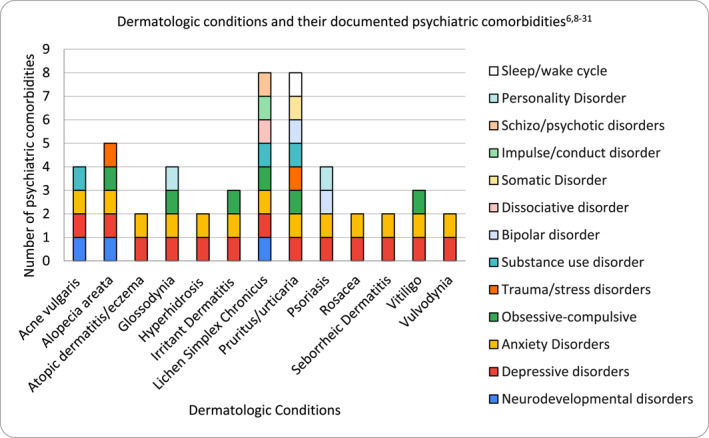

1

**What is already known about this topic?**

- These psychocutaneous disorders have definitions and established treatments in both dermatology and psychiatry. The knowledge appears to be psychocentric and/or dermatologically skewed within each specialty.

**What does this study add?**

- The intersection of these diseases have not been well studied nor do dermatologists or psychotherapists have a deep understanding of each other's concept of these diseases. After review of the literature, we were able to come up with surprising connections that were not previously well understood. We hope this will serve as a bridge between dermatology and psychiatry.

## 2 INTRODUCTION

A psychocutaneous disorder is a condition that afflicts the mind and the skin simultaneously.^1^ Psychocutaneous disorders are a unique intersection of two specialized fields of study. There have been many published papers reviewing psychocutaneous disorders, however, this review provides a cursory, but standardized perspective in which to assist clinicians to comprehend and integrate the current perspectives and treatments related to each disorder from a psychotherapeutic perspective. Our aim is to clarify and simplify the approach to treatment while providing pertinent background information on the aetiology and pathophysiology.

30% of patients with dermatologic disorders have a psychiatric comorbidity.^2^ Hence, it is crucial to treat underlying psychiatric conditions that might precipitate or exacerbate dermatologic conditions. The psychiatric comorbidities in the literature are depressive disorders, anxiety disorders, and obsessive compulsive related disorders.^3^ In many cases, it is unclear if a psychiatric disorder precipitated a dermatologic manifestation or if a dermatologic disorder catalyzed a psychiatric disorder. Dermatologic conditions have significant interplay with primary psychiatric conditions like depression and anxiety. Significant distress from dermatologic conditions is capable of inducing or exacerbating psychiatric illness. For example, although isotretinoin has a blackbox warning for the potential to induce depression and suicidal thoughts in adolescents, a particular study found that treatment of acne using isotretinoin therapy reduced Beck Depression Inventory scores from 9.77 to 5.86 (*p* = 0.001).^4^, ^5^

A condition that illuminates the intersection of psychiatry and dermatology is lichen simplex chronicus. In this condition, there can be an underlying primary dermatologic condition such as eczema or atopic dermatitis. In this case, the patient may repetitively scratch to relieve symptoms. Serotonin released in the central nervous system during scratching gives a momentary pleasurable sensation which ultimately reinforces the maladaptive behaviour.^6^, ^7^ This presents as thickened areas of skin which can progress to cover large areas of the body and become disfiguring. At this junction, both the dermatologist and mental health provider should understand the underlying psychiatric comorbidities.

The topics we discuss have been broken down into two categories: dermatologic disorders with psychiatric manifestations and psychiatric disorders with dermatologic manifestations. We plan to briefly define each of the major dermatologic and psychocutaneous disorders as well as the associated pathophysiology. We will provide a table of psychocutaneous disorders which have specific psychotherapeutic treatments, as well as a figure demonstrating the most common psychiatric comorbidities. Of note, we have only included the most common psychocutaneous disorders seen by dermatology in this review and excluded less common psychocutaneous disorders. We are also excluding discussion on dermatologic management as a dermatologist would already be well‐versed.

## 3 METHODS

This literature search was conducted by primarily using the research platforms of pubmed, University of Maryland (UMD) library OneSearch, and Towson University's Cook One Search. The OneSearch platform has access to 49 EBSCO databases which are viewable on TU and UMD's library websites. The national centre for biotechnology information (NCBI), wiley online library, and scopus were also utilized. We then reviewed the reference lists of sources to identify additional relevant studies. When using the UMD and TU library OneSearch, ‘full text articles’ and ‘peer reviewed’ were selected to only show full text, peer reviewed articles in the search results. Each of the psychocutaneous disorders were searched separately and each disorder was searched several ways. The first way would include: ‘condition name (acne vulgaris, alopecia areata, atopic dermatitis, glossodynia, hyperhidrosis, irritant dermatitis, lichen simplex chronicus, pruritus, psoriasis, rosacea, seborrhoeic dermatitis, urticaria, vitiligo, vulvodynia, body dysmorphic disorder (BDD), dermatitis artefacta, delusions of parasitosis, eating disorders, bulimia nervosa, anorexia nervosa, morgellons, acne excoriée, dermatillomania, onychotillomania, or trichotillomania)’ AND ‘treatment or therapy or intervention’. The second way would include: ‘condition name’ AND ‘psychiatry or psychiatric or mental health’. The third way would include: ‘condition name’ AND ‘anxiety and depression’. Each condition was searched in these 3 ways to generate articles that would most comprehensively cover the psychiatric treatment, dermatologic treatment, and psychiatric comorbidities of each condition. The title and abstract of each article were screened for relevancy. Selected articles were then reviewed by the individual researcher that chose the article and relevant data was extracted and incorporated into this review. Articles written before 2000 or in a language other than English were excluded. Articles that were peer reviewed or had full text access were included. The review was conducted from 21 February 2022 through 6 June 2022. Four researchers participated in the search process.

## 4 RESULTS

**FIGURE 1:**
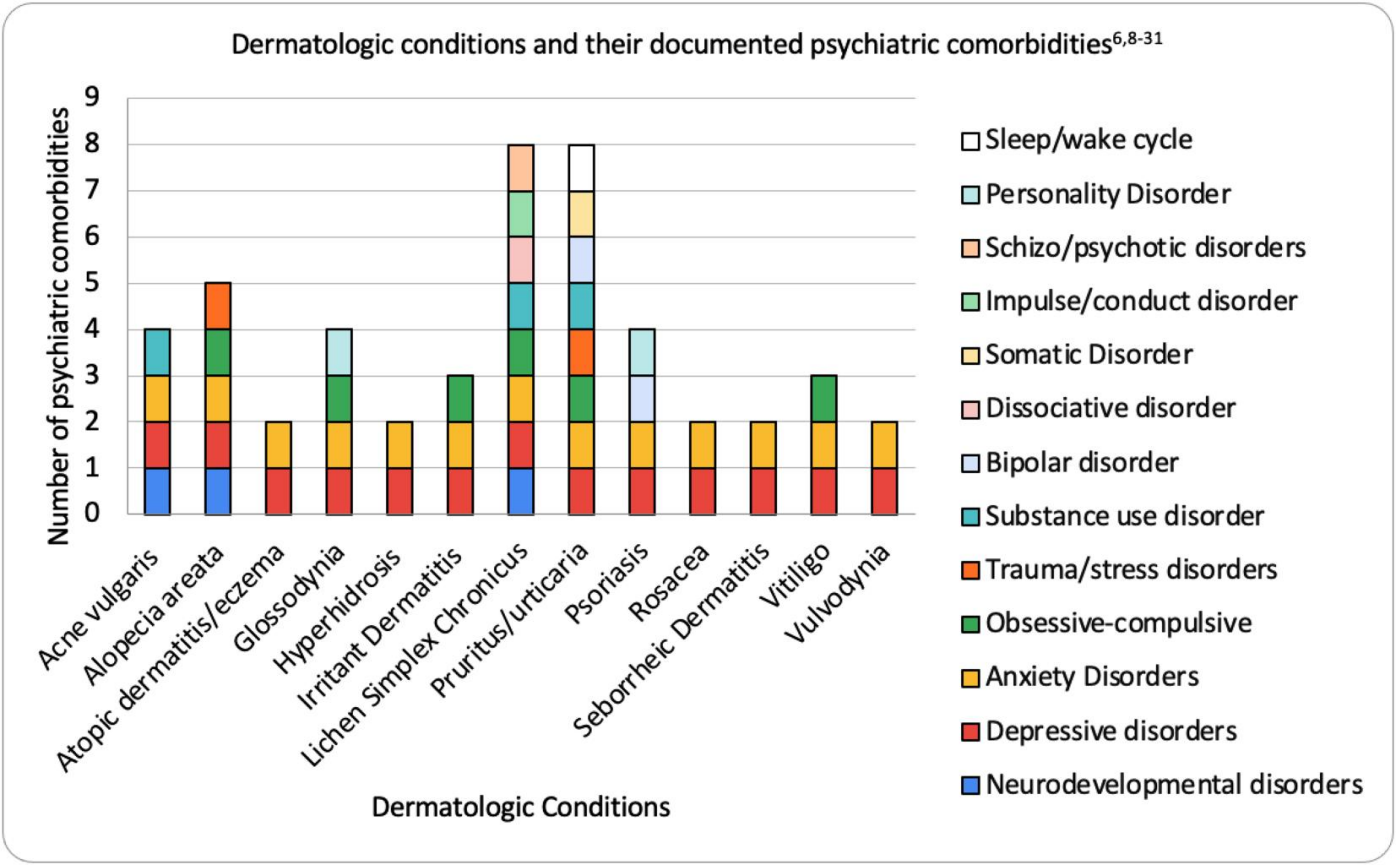
Dermatologic conditions and their documented psychiatric comorbidities.

The common primary dermatologic disorders and each disorder's documented psychiatric comorbidities have been illustrated (Figure 1). Table 1 shows the primary psychiatric disorders, psychotherapeutic treatment, and common psychiatric comorbidities. For better understanding, we have provided the definitions of the most common primary dermatologic disorders followed by the primary psychiatric disorders. Included in this is the definition, followed by the pathophysiology, followed by the treatment.

**TABLE 1:** Psychiatric disorders with dermatologic manifestations^6^, ^9^, ^32^, ^33^, ^34^, ^35^, ^36^, ^37^, ^38^, ^39^, ^40^, ^41^, ^42^, ^43^, ^44^, ^45^, ^46^, ^47^, ^48^, ^49^, ^50^, ^51^, ^52^, ^53^, ^54^, ^55^, ^56^, ^57^, ^58^, ^59^, ^60^, ^61^, ^62^, ^63^, ^64^, ^65^, ^66^

Psychiatric disorders with dermatologic manifestations	Psychotherapeutic approach	Most common psychiatric comorbidities
BDD	Psychotherapy (CBT, behaviour modification)	Depressive disorders, anxiety disorders, psychotic disorders, OCD, substance use disorders, avoidant personality disorder, attention deficit/hyperactivity disorders
	Medications: SSRIs (fluoxetine, escitalopram), TCAs (clomipramine), MAOIs, and pimozide	
Dermatitis artefacta (factitial dermatitis)	Psychotherapy (CBT, dialectical behavioural therapy, and relaxation therapy)	Depressive disorders, anxiety disorders, BPD, substance use disorders, eating disorders, somatic symptom disorders, Munchausen syndrome
	Medications: SSRIs, anxiolytics, antipsychotics	
Psychogenic excoriation: acne excoriee, dermatillomania, onychotillomania, trichotillomania	Psychotherapy (CBT, habit reversal, dialectical, psychodynamic insight‐oriented, acceptance‐enhanced), TMS, biofeedback, self‐monitoring, desensitization, hypnosis	Depressive disorders, anxiety disorders, OCD, personality disorders, substance use disorder, BDD, eating disorders, bipolar disorders, attention deficit/hyperactivity disorders, schizophrenia, dissociative disorders, oppositional defiant disorder, elimination disorders, tic disorders
	Medications: antihistamines, SSRIs (fluoxetine, fluvoxamine, sertraline, citalopram), TCAs (clomipramine, desipramine), SNRIs (venlafaxine), antipsychotics (olanzapine, paliperidone, aripiprazole, pimozide), anxiolytics (lorazepam, clonazepam, buspirone), atypical antidepressants (trazodone), anticonvulsants (oxcarbazepine), lithium, naltrexone, N‐acetylcysteine, lamotrigine	
Delusional disorder: delusions of parasitosis, morgellons	Psychotherapy: CBT, habit reversal, or insight oriented therapy; transcranial magnetic stimulation (TMS) supplemental therapies: Yoga, meditation, mindfulness, aerobic exercise, relaxation techniques, journaling, hypnosis, acupuncture, and biofeedback	Depressive disorders, anxiety disorders, OCD, personality disorders, substance use disorder, BDD, and eating disorders
	Medications: second generation antipsychotics (olanzapine, risperidone, quetiapine, and aripiprazole)	
	First generation antipsychotics: pimozide	
Eating disorders: bulimia nervosa, anorexia nervosa	Family‐based treatment (Maudsley method), cognitive behavioural therapy, interpersonal psychotherapy, group therapy mindfulness (meditation and yoga)	OCD, obsessive‐compulsive personality disorder, social phobia, anxiety disorders, substance use disorders, and personality disorders major depression, impulse control disorders, mood disorders
	Atypical antipsychotics (olanzapine)	
	SSRIs	

Abbreviations: BDD, body dysmorphic disorder; BPD, borderline personality disorder; CBT, cognitive behavioural therapy; MAOI, mono‐amine oxidase inhibitor; OCD, obsessive‐compulsive disorder; SNRI, serotonin norepinephrine reuptake inhibitor; SSRI, selective serotonin reuptake inhibitor; TCA, tricyclic antidepressant; TMS, transcranial magnetic stimulation.

### 4.1 Dermatologic disorders with psychiatric manifestations

#### 4.1.1 Acne vulgaris

Acne vulgaris occurs due to increased sebum production, hyperkeratinization leading to blockage of the pilosebaceous unit, colonization of bacteria, and inflammation of the skin.^67^, ^68^, ^69^ Psychotherapeutic treatment focuses on psychiatric comorbidities that were already present or that arise as a result of the condition. Treatment also involves treating the underlying acne which requires a team approach with a dermatologist. Common psychiatric comorbidities include depressive disorders, anxiety disorders, attention deficit/hyperactivity disorder, and substance use disorder.^8^ Non‐pharmacologic therapies include psychotherapy and supplemental therapies such as yoga, meditation, mindfulness, aerobic exercise, relaxation techniques, journaling, hypnosis, acupuncture, and biofeedback.^9^, ^70^ Pharmacologic therapy for comorbid depression and anxiety includes selective serotonin reuptake inhibitors (SSRIs), serotonin and norepinephrine reuptake inhibitors (SNRIs), tricyclic antidepressants (TCAs), monoamine oxidase inhibitors (MAOIs), atypical antidepressants, and anxiolytics.^3^, ^71^

An example of treatment would be to take baseline photographs and at periodic intervals to show objective improvement to the patient, keeping in mind that many of these patients are adolescents and tend to be nonadherent. Written or verbal contracts between the provider and patient could be employed to enhance compliance.^72^

#### 4.1.2 Alopecia areata

Alopecia areata is an autoimmune condition in which hair follicles are subject to an inflammatory response that inhibits the growth of the follicle, resulting in transient bouts of hair loss and the clinical presentation of well defined, hairless patches on hair‐bearing parts of the body, most commonly the scalp.^73^, ^74^, ^75^

Treatment again involves treating any underlying psychiatric comorbidities and those that arise as a result of the condition, as well as restoration of self‐image and confidence. Common psychiatric comorbidities include depressive disorders, anxiety disorders, attention deficit/hyperactivity disorders, obsessive compulsive disorders, and adjustment disorder.^10^, ^11^ Treatment may involve the use of psychotherapy, supplemental therapies such as yoga, meditation, mindfulness, aerobic exercise, relaxation techniques, journaling, hypnosis, acupuncture, and biofeedback, SSRIs, SNRIs, TCAs, MAOIs, atypical antidepressants, and anxiolytics.^3^, ^5^, ^6^, ^71^, ^76^

The use of mindfulness therapy in the form of stress reduction for instance, meditation, could help the patient cope while other ongoing standard pharmacologic therapies are being administered.^77^

#### 4.1.3 Atopic dermatitis

##### Other names: Eczematous dermatitis or atopic eczema

Atopic dermatitis is a complex, multimodal, chronic inflammatory skin condition where genetic predispositions, immune system dysregulation, and decreased microbial diversity leads to disruption of the epidermal barrier and subsequent skin dehydration, pruritus, inflammation, and other complications.^78^, ^79^

Affected patients tend to be more anxious and depressed than those without atopic dermatitis.^6^, ^12^ Anxiety and depression can amplify itch perception and provoke scratching behaviour.^6^ Non‐pharmacological psychotherapeutic treatment includes psychotherapy such as brief dynamic or cognitive behavioural therapy (CBT) and supplemental therapies such as yoga, meditation, mindfulness, aerobic exercise, relaxation techniques, journaling, hypnosis, acupuncture, and biofeedback.^6^, ^71^, ^80^ Psychotropic drugs with promising evidence to reduce itch in atopic dermatitis include TCAs (doxepin and trimipramine), bupropion, naltrexone, SSRIs (paroxetine and fluvoxamine), and anxiolytics (tandospirone citrate). Antihistamines may also be initiated.^80^

Patient education regarding the pathophysiology of the disease with transepidermal water loss is critical to achieving better therapeutic outcomes. Simple but thorough handouts listing the use of proper moisturization therapy and avoidance of contact irritants should go very far in achieving therapeutic endpoints. In the meantime, any form of stress reduction can reduce the pruritus and break the itch scratch cycle.^81^, ^82^ Patients with large body surface involvement may need to be on a biologic in order to alleviate their symptoms. The use of pharmacologic products should still be complemented with stress reduction and adherence to topical emollients.^83^, ^84^

#### 4.1.4 Glossodynia

##### Other name: Burning mouth syndrome

Glossodynia is an oral pain syndrome characterized by a burning sensation on the tongue in the absence of physical trauma, gross lesions, or other oral pathology potentially accompanied by the symptoms of dry mouth, altered taste perception, or halitosis. The pathophysiology of this disorder is poorly understood and is thought to arise from a hypoactive chorda tympani division of the trigeminal nerve, resulting from trauma or other degenerative processes.^85^ Altered cortical connectivity originating from trigeminal afferent signals induces central sensitization that may account for the burning sensation. Systemic factors such as disruptions in hormonal balance, dysregulation of the hypothalamic pituitary axis, and psychogenic factors like chronic generalized anxiety may also contribute to this disorder.^86^

Although there is no definitive cure, the condition can spontaneously resolve. Additionally, there are different medication classes that may be tried by the dermatologist to alleviate symptoms. Medications include antidepressants such as paroxetine, anxiolytics such as benzodiazepines including oral or topical clonazepam, topical local anaesthetics, analgesics, alpha‐lipoic acid, and topical capsaicin.^87^

One approach focuses on actively listening and reassuring the patient that there are no severe underlying medical conditions precipitating symptoms, in addition to identifying and implementing healthy coping mechanisms during periods of increased stress.^13^ Providers must first ensure medical aetiologies are ruled out before providing reassurance which may entail referrals to other specialists and providers. Treatment includes psychotherapy such as CBT and supplemental therapies such as yoga, meditation, mindfulness, aerobic exercise, relaxation techniques, journaling, hypnosis, acupuncture, and biofeedback are beneficial.^13^, ^71^, ^86^ Pharmacologic treatment may include antidepressants such SSRIs, SNRIs, TCAs, MAOIs, and atypical antidepressants, as well as anxiolytics.^3^, ^72^, ^86^ Common psychiatric comorbidities include depressive disorders, anxiety disorders, personality disorders, and obsessive compulsive disorders.^13^, ^87^

#### 4.1.5 Hyperhidrosis

Hyperhidrosis is defined as persistent, chronic, and excessive localized or generalized secretion of sweat from sudoriferous glands of the glabrous skin and axilla that is independent of thermal homoeostatic regulation.^88^, ^89^, ^90^, ^91^ Treatment focuses on treating the underlying depressive disorders and anxiety disorders that precipitate primary hyperhidrosis.^92^, ^93^ Non‐pharmacologic therapy includes psychotherapy and supplemental therapies such as acupuncture and reflexology.^94^, ^95^ Other supplemental therapies such as yoga, meditation, mindfulness, aerobic exercise, relaxation techniques, journaling, hypnosis, and biofeedback may also be helpful for concurrent depression or anxiety.^9^ Pharmacologic treatment may include antidepressants such SSRIs, SNRIs, TCAs, MAOIs, and atypical antidepressants, as well as anxiolytics.^3^, ^71^

#### 4.1.6 Irritant dermatitis

Irritant dermatitis is a non‐immunologically related physical or chemical insult to regions of the skin, resulting in burning, pruritus, erythema, and inflammation. Irritants are numerous and may include detergents, friction, or extremes in temperature. The psychotherapeutic approach again revolves around treating the psychiatric comorbidities that give rise to the condition or that arise due to the condition. The common psychiatric comorbidities include depressive disorders, anxiety disorders, and obsessive compulsive disorders.^14^, ^15^ Therapies that may be used for these conditions include psychotherapy, supplemental therapies such as yoga, meditation, mindfulness, aerobic exercise, relaxation techniques, journaling, hypnosis, acupuncture, and biofeedback, oral medications such as SSRIs, SNRIs, TCAs, MAOIs, atypical antidepressants, and anxiolytics.^3^, ^6^, ^9^, ^73^

#### 4.1.7 Lichen simplex chronicus

Lichen simplex chronicus is the lichenification of skin characterized by circumscribed, erythematous, thick, and scaly plaques and patches with defined skin lines as the result of repeated excoriations due to psychogenic pruritus, psychiatric comorbidities, or arising secondary to pruritic cutaneous disorders. This disorder is heavily documented with psychiatric comorbidities. The most common comorbidities include depressive disorders, especially major depression and dysthymic disorder; anxiety disorders, attention deficit/hyperactivity disorder, substance use disorder, psychotic disorders, dissociative disorders, obsessive compulsive disorders, conduct disorder, and impulse‐control disorders.^16^, ^17^, ^18^ The psychiatric treatment revolves around treating the psychiatric comorbidities. Psychotherapy, especially CBT, and supplemental therapies such as yoga, meditation, mindfulness, aerobic exercise, relaxation techniques, journaling, hypnosis, acupuncture, and biofeedback may be helpful.^6^, ^9^, ^96^ Pharmacologic therapy may include SSRIs, SNRIs, TCAs, MAOIs, atypical antidepressants, antipsychotics, and anxiolytics, depending on the condition being treated.^3^, ^71^

One example to treat these patients would be to employ aspects of CBT, such as mindfulness, by having the patient place masking tape on their vanity mirror and writing on the tape ‘The Doctor is watching, no picking’. This blatantly and painstakingly makes the patient aware on the continual basis that they are doing harmful, repetitive behaviour that should be halted.^97^

#### 4.1.8 Pruritus

##### Includes generalized, localized, and psychogenic pruritus

Pruritus is defined as cutaneous itching sensations that can have primary and secondary causes, can be localized or systemic, and can be spontaneous, chronic, or relapsing. Pruritus can be neuropathic, neurogenic, psychogenic, integumentary, or mixed in nature.^98^ Mediators of itching sensation include histamine, serotonin, kinin release, mu opioid receptors in neurogenic pruritus, endocannabinoids, prostaglandin release, and activation of afferent C‐fibres.^98^, ^99^ The psychiatric approach revolves around treating the comorbidities which are most commonly depressive disorders, anxiety disorders, adjustment disorder, substance use disorder, post‐traumatic stress disorder, and somatic symptom related disorders.^19^ Treatment may entail psychotherapy or supplemental therapies such as yoga, meditation, mindfulness, aerobic exercise, relaxation techniques, journaling, hypnosis, acupuncture, and biofeedback.^6^, ^9^ Treatment may also entail systemic medications such as SSRIs, SNRIs, TCAs, MAOIs, atypical antidepressants, anxiolytics, and antipsychotics depending on the condition being treated.^3^, ^6^, ^9^, ^71^

#### 4.1.9 Psoriasis

Psoriasis is a chronic inflammatory disorder characterized by annular, well defined, scaling plaques, with or without pruritus, formed from epidermal hyperplasia and reduced migration time of keratinocytes to the stratum corneum. The most common psychiatric comorbidities with psoriasis are depressive disorders, anxiety disorders, mood disorders, and various personality disorders, specifically schizoid, avoidant, passive‐aggressive, and obsessive compulsive personality disorders.^6^, ^20^, ^21^, ^22^ Treatment may entail psychotherapy, supplemental therapies such as yoga, meditation, mindfulness, aerobic exercise, relaxation techniques, journaling, hypnosis, acupuncture, and biofeedback. Treatment may also entail psychotropic drugs such as SSRIs, SNRIs, TCAs, MAOIs, atypical antidepressants, anxiolytics, mood stabilizers, and antipsychotics depending on the condition being treated.^3^, ^9^, ^71^

#### 4.1.10 Psychogenic purpura syndrome

##### Other names: Gardner‐diamond syndrome, autoerythrocyte sensitization syndrome

Psychogenic purpura syndrome is a rare condition characterized by spontaneous oedema and subsequent ecchymosis that occurs within a 24‐h window after severe acute stressors or trauma.^23^ Patients present with isolated or multiple painful oedematous lesions progressing to bluish ecchymosis with a yellowish hue primarily located on the extremities. Psychotherapy and the placebo effect have produced positive results.^23^, ^24^

The most common psychiatric comorbidities are depressive disorders, anxiety disorders, illness anxiety disorder, borderline personality disorder (BPD), histrionic personality disorder, bipolar disorder, conversion disorder, and obsessive compulsive disorders.^23^, ^24^ SSRIs are the most effective treatment for this condition. Psychotherapy such as CBT is also recommended and is most successful in combination with a SSRI. Other medication classes such as antipsychotics, anxiolytics, and other antidepressants may be used to treat comorbid conditions.^24^, ^25^, ^26^, ^27^, ^28^, ^29^, ^30^, ^31^, ^32^, ^33^, ^34^, ^35^, ^36^, ^37^, ^38^, ^39^, ^40^, ^41^, ^42^, ^43^, ^44^, ^45^, ^46^, ^47^, ^48^, ^49^, ^50^, ^51^, ^52^, ^53^, ^54^, ^55^, ^56^, ^57^, ^58^, ^59^, ^60^, ^61^, ^62^, ^63^, ^64^, ^65^, ^66^, ^67^, ^68^, ^69^, ^70^, ^71^, ^72^, ^73^, ^74^, ^75^, ^76^, ^77^, ^78^, ^79^, ^80^, ^81^, ^82^, ^83^, ^84^, ^85^, ^86^, ^87^, ^88^, ^89^, ^90^, ^91^, ^92^, ^93^, ^94^, ^95^, ^96^, ^97^, ^98^, ^99^, ^100^, ^101^, ^102^

#### 4.1.11 Rosacea

##### Includes erythematotelangiectatic, papulopustular, ocular, phymatous, and rhinophyma

Rosacea is a chronic inflammatory disorder characterized by erythema, telangiectasia, eruption of papules and pustules, or phymatous changes affecting the cheeks, nose, chin, eyes, or forehead.^103^ The most common psychiatric comorbidities are depressive disorders and anxiety disorders.^8^, ^25^ Treatment for these conditions may entail psychotherapy or supplemental therapies such as yoga, meditation, mindfulness, aerobic exercise, relaxation techniques, journaling, hypnosis, acupuncture, and biofeedback. Treatment may also entail psychotropic drugs such as SSRIs, SNRIs, TCAs, MAOIs, atypical antidepressants, and anxiolytics.^3^, ^9^, ^71^

#### 4.1.12 Seborrhoeic dermatitis

Seborrhoeic dermatitis is a chronic inflammatory disorder characterized by scaling, greasy, erythematous macules on sebaceous regions of the scalp, face, ears, chest, and back. Genetics, psychological stress, food sensitivities, and the changing of seasons can also contribute.^104^, ^105^

The most common psychiatric comorbidities are depressive disorders and anxiety disorders.^26^, ^27^ Treatment for these conditions may entail psychotherapy or supplemental therapies such as yoga, meditation, mindfulness, aerobic exercise, relaxation techniques, journaling, hypnosis, acupuncture, and biofeedback. Treatment may also entail psychotropic drugs such as SSRIs, SNRIs, TCAs, MAOIs, atypical antidepressants, and anxiolytics.^3^, ^9^, ^71^

#### 4.1.13 Urticaria

##### Chronic idiopathic urticaria

Urticaria is a systemic IgE autoantibody reaction that catalyzes spontaneous flaring with resulting pruritic wheals and welts. Triggers of urticaria may include high stress, exercise, food sensitivities, sun exposure, and more.^106^, ^107^, ^108^ The most common psychiatric comorbidities are depressive disorders, mood disorders, anxiety disorders, sleep and wakefulness disorders, trauma and stressor disorders, somatic disorders, obsessive compulsive disorders, and substance use disorders.^28^ Treatment may entail psychotherapy, especially CBT, or supplemental therapies such as yoga, meditation, mindfulness, aerobic exercise, relaxation techniques, journaling, hypnosis, acupuncture, and biofeedback. Treatment may also entail psychotropic drugs such as SSRIs, SNRIs, TCAs, MAOIs, atypical antidepressants, anxiolytics, and antipsychotics depending on the condition being treated.^3^, ^9^, ^28^, ^71^

#### 4.1.14 Vitiligo

Vitiligo is a dermatologic disorder characterized by diffuse depigmented patches of skin that often arise with idiopathic origins. It is considered both a genetic and autoimmune disorder.^109^ The pathophysiology of vitiligo involves defects in melanocytic cells combined with high oxidative stress in the microenvironment resulting in local melanocyte destruction.^109^, ^110^

The most common psychiatric comorbidities are depressive disorders, OCD, and anxiety disorders, especially social phobia and panic disorder.^28^ Treatment may entail psychotherapy, or supplemental therapies such as yoga, meditation, mindfulness, aerobic exercise, relaxation techniques, journaling, hypnosis, acupuncture, and biofeedback. CBT, psychoeducation, and exposure/response prevention therapies are especially helpful for OCD comorbidity.^6^ Treatment may also entail psychotropic drugs such as SSRIs, SNRIs, TCAs, MAOIs, atypical antidepressants, anxiolytics, and antipsychotics depending on the condition being treated.^3^, ^9^, ^71^

#### 4.1.15 Vulvodynia

Vulvodynia is characterized by chronic pain in the external aspects of the female genitalia that is present for at least 3 months and has no readily identifiable aetiology.^111^ Pathophysiology is poorly understood but likely due to central sensitization of the region, or unidentifiable peripheral nerve injury to any aspect of the female external genitalia. The result is hyperactivation of nociceptive fibres and subsequent sensation of pain and dysaesthesia.^30^, ^112^

A dermatologist can suggest avoiding irritants and a gentle regimen for proper vulvar hygiene and hydration.^111^ Treatment may entail behaviour modification, pelvic floor exercises or physical therapy, cool packs, topical local anaesthetics, topical oestrogen, topical testosterone, nerve blocks, botox injections, topical amitriptyline, topical corticosteroids, and surgery such as a vestibulectomy.^112^, ^113^ Systemic therapies include TCAs such as amitriptyline, nortriptyline, doxepin, or desipramine; SNRIs such as venlafaxine or duloxetine; and anticonvulsants such as gabapentin, pregabalin, lamotrigine, topiramate, or carbamazepine.^112^, ^113^

Vulvodynia is highly comorbid with psychiatric and other chronic pain conditions, as well as occurring more frequently in those with a history of sexual abuse. Patients are 4× more likely to be diagnosed with a history of a depressive or anxiety disorder.^30^, ^31^ Psychotherapy, especially CBT and supplemental therapies such as yoga, meditation, mindfulness, aerobic exercise, relaxation techniques, journaling, hypnosis, acupuncture, and biofeedback are beneficial.^8^, ^9^, ^110^, ^111^, ^112^, ^113^ Pharmacologic therapy includes the antidepressants listed above as well as the serotonin norepinephrine reuptake inhibitor milnacipran and antipsychotics.^71^ Other medication classes that may be used include SSRIs, MAOIs, atypical antidepressants, and anxiolytics for potential comorbid depression or anxiety.^3^, ^71^

### 4.2 Psychiatric disorders with dermatologic manifestations

The *Diagnostic and Statistical Manual of Mental Disorders* is comprised of the official nomenclature and classifications used by mental health professionals.^32^ The most recent edition, the fifth edition, contains 22 major categories of mental illnesses. Listed below, in table format, are the 22 major categories with minor categories which are relevant to this paper. All irrelevant minor categories were excluded.

#### 4.2.1 Body dysmorphic disorder

##### Other name: Dysmorphophobia

BDD is a chronic psychiatric disorder characterized by the imagination or magnification of defects which are insignificant or not apparent to others and causing significant distress or impairment in functioning.^6^ The pathophysiology is poorly understood, but deficits in facial processing regarding excessive activation of the dorsal cingulate cortex, which is activated in response to error processing, and left hemispheric lateralization is thought to convey excessive detail and analytic information.^114^ This excessive detail and relative inability to see the configural formation of the body or face leads to excessive awareness of imperfections.^114^ BDD is classified under obsessive compulsive and related disorders in the DSM‐5.^32^

Treating BDD with medical and surgical procedures is almost always unsuccessful and furthermore may lead to a malpractice‐suit.^6^ The dermatologist must be careful about approaching the BDD patient and must first build proper rapport. Patients are generally reluctant to accept psychiatric help and suggesting such before proper rapport is established may be detrimental to the patient provider relationship. The dermatologist may employ treatment with an antidepressant if they feel comfortable, or attempt to refer the patient to a mental health professional for more involved treatment.^33^

Psychotropic drugs that may be useful include SSRIs, especially fluoxetine or escitalopram, TCAs, especially clomipramine, MAOIs, and pimozide. Augmenting a SSRI with clomipramine, buspirone, lithium, methylphenidate, or antipsychotics may have a better response.^6^, ^34^ Other comorbid psychiatric conditions are common and should be treated appropriately. The most common psychiatric comorbidities are depressive disorders, anxiety disorders, especially social phobia; psychotic disorders, obsessive compulsive disorders, substance use disorders, avoidant personality disorder, and attention deficit/hyperactivity disorders.^6^, ^35^, ^36^ Psychotherapy such as CBT therapy may be especially helpful.^14^

One example in treating BDD is to employ aspects of CBT to teach the patient that they have a heightened awareness of their perceived imperfections of the skin and help them realize that they have a higher awareness compared to the general population which leads to their discomfort. An example that one can employ in the office for a patient that complains of an imperceivable wrinkle is to show them photographs of another patient with truely photodamaged skin highlighting the actual wrinkles. This will be a reality test for the patient that is hard evidence that should alleviate their immediate anxiety.^37^

#### 4.2.2 Dermatitis artefacta

##### Other names: Factitial dermatitis, dermatitis factitia, dermatitis simulata

Dermatitis artefacta is a factitious disorder characterized by self induced skin lesions in which methods beyond simple excoriation are used to create the lesions and elicit sympathy or medical attention. Patients will often provide a vague history and timeline of the lesions. A variety of skin lesions can be observed such as blisters, ulcers, erythema, oedema, purpura, or sinuses, and the morphology of the skin lesions is often bizarre, linear, or geometric with healthy, unaffected skin bordering the lesions. Lesions will be found in areas accessible to the patient such as extremities, face, and anterior trunk.^38^, ^39^, ^40^ Dermatitis artefacta is classified as a factitious disorder under somatic system and related disorders in the DSM‐5.^32^

The initial approach the dermatologist may take is a skin biopsy to rule out dermatological causes as dermatitis artefacta is a diagnosis of exclusion. Spectrometry may be done to identify foreign material in the lesions.^38^, ^39^ Treatment of the lesions includes debridement, irrigation, occlusive bandaging, emollients, topical antibiotics, topical steroids, topical antifungals, and systemic antibiotics or antifungal medications.^39^, ^40^, ^41^

The psychiatric approach involves both behavioural therapy and psychotropic medications. CBT, dialectical behavioural therapy, and relaxation therapy may be particularly efficacious.^39^, ^40^, ^41^, ^42^ SSRIs such as fluoxetine, sertraline, paroxetine, and fluvoxamine are first line for compulsive behaviours. Anxiolytics such as buspirone and benzodiazepines may be used for comorbid anxiety. Antipsychotics such as pimozide, olanzapine, and risperidone may be helpful for self‐injurious behaviours and can be combined with a SSRI. Common psychiatric comorbidities include depressive disorders, anxiety disorders, BPD, substance use disorders, eating disorders, somatic symptom disorders, and Munchausen syndrome.^38^, ^39^, ^40^, ^41^

#### 4.2.3 Eating disorders

##### Anorexia nervosa and bulimia nervosa

Anorexia nervosa involves restricting food intake, an intense fear of gaining weight, and a disturbed self‐image that results in low body weight over a period of at least 3 months. Anorexia nervosa can be broken down into two categories: restricting type and binge‐eating/purging type. The restricting subtype describes weight loss in the last 3 months that is achieved by dieting, fasting, and/or excessive exercise. The binge‐eating/purging subtype describes recurrent episodes of binge eating or purging behaviour. Examples of purging behaviour include self‐induced vomiting, laxative use, enemas, and diuretics.^32^

The pathophysiology of anorexia nervosa is complex and multifactorial. Genetic factors have recently been revealed in a twin study that demonstrated high inheritability.^115^ There is evidence of an interaction between these genetic risks and other identified risk factors such as anxiety, perfectionism, cognitive rigidity, and early feeding difficulties.^116^ As understanding of the genetics of anorexia nervosa builds, it has been proposed that the significant genetic correlations with psychiatric disorders, physical activity and metabolic traits deserves viewing anorexia nervosa as a ‘metabo‐psychiatric disorder’.^117^ In contrast with bulimia nervosa, sexual abuse is not a risk factor for anorexia nervosa.^118^ Although a psychiatric disorder, around half of deaths are attributable to physical complications associated with starvation.

Bulimia nervosa involves the uncontrolled consumption of an abnormally large amount of food within a 2‐h window and followed by overcorrecting with self‐induced vomiting, laxatives, fasting, or excessive exercise accompanied by a sense of lack of control. The frequency of this behaviour occurs at least once a week for 3 months to make the diagnosis.^32^

The majority of cases of bulimia nervosa identified in a national community survey featured were associated with an additional psychiatric disorder.^119^ The common comorbidities were anxiety, mood, impulse control, and substance‐use disorders. The aetiology of bulimia nervosa is also complex, but sociocultural pressures to be thin and the promotion of dieting seem to increase risk. One community‐based case‐control study found higher rates of obesity, mood disorder, sexual and physical abuse, parental obesity, substance misuse, low self‐esteem, perfectionism, disturbed family dynamics, parental weight/shape concern, and early menarche in people with the bulimia nervosa. Heritability is high with bulimia nervosa as well, ranging from 28% to 83%.^43^

Eating disorders primarily affect those ages 12–25 years of age and can present with a spectrum of severity ranging from a healthy weight with subtle symptoms to requiring inpatient care for electrolyte imbalances and heart failure.^43^, ^119^ If an eating disorder is suspected, a thorough history should include questions about the rate and amount of weight loss, compensatory behaviours, exercise, menstrual history and questions to identify physical symptoms of underweight or weight loss, such as cold intolerance, fatigue, dizziness or fainting, chest pain and palpitations.^120^

There are many skin signs of eating disorders and recognizing these skin manifestations may reveal an undiagnosed eating disorder. The skin manifestations can impact the skin, oral cavity, hair, and nails and are results of starvation, medication side effects, vomiting, and other accompanying psychiatric illnesses.^121^ The most common skin manifestations include asteatotic skin and follicular hyperkeratosis, carotenoderma, hyperpigmentation, acrocyanosis and perniosis, acne, and pruritus. Less common skin signs include striae distensae, seborrhoeic dermatitis, diffuse reticulate purpura, pellagra, scurvy, acquired acrodermatitis enteropathica, and erythema ab igne.^121^ The hair and nail changes include lanugo‐like body hair, alopecia, opaque hair, pili torti, and nail fragility. Signs within the oral cavity include angular cheilitis, gingivitis, and taste abnormalities.^121^ The signs that are specific to eating disorders include lanugo‐like body hair from self‐imposed starvation, Russel sign (knuckle calluses) and enamel erosion from self‐induced vomiting, and self‐induced dermatoses from co‐morbid psychiatric conditions.^121^ These signs are not reported in starvation caused by famine, protein energy malnutrition, marasmus, Kwashiorkor, or vitamin C and K deficiencies.^121^

A current or past episode of major depression has been reported in more than half of patients with eating disorders.^44^ Other associated psychiatric disorders include obsessive‐compulsive disorder (OCD), obsessive‐compulsive personality disorder, social phobia, anxiety disorders, substance use disorders, and personality disorders.^32^ Psychological symptoms include heightened emotional arousal, reduced tolerance of stress, emotional dysregulation, social withdrawal, and self‐critical perfectionistic traits.^32^ Suicidal ideations have been found to be associated with anorexia nervosa.^122^

Aside from the nutritional goal of restoring 90% of the patient's age appropriate body weight with nutritional support, there are several psychological interventions:

Psychotherapy is the key to successful treatment of an eating disorder. The goals of psychotherapy include reduction of distorted body image and dysfunctional eating habits, return to social engagement, and resumption of full physical activities.^45^ Family‐based treatment, also known as the Maudsley method, is a promising method for adolescents who have bulimia nervosa.^43^ CBT and interpersonal psychotherapy have been shown in clinical trials to significantly improve those with bulimia nervosa.^46^ Group therapy has also been incorporated into eating disorder treatment programs.^47^ Mindfulness practices such as meditation and yoga benefit those with underlying anxiety.^48^

Medications play a role in treating comorbid psychiatric conditions but should not be used alone in the treatment of eating disorders. Studies have shown limited evidence for medications in the treatment of anorexia nervosa. Antidepressants such as SSRIs may help with underlying depression but they have not been shown to improve weight gain or prevent relapse in those with anorexia nervosa.^49^, ^50^ Case reports have suggested effectiveness of atypical antipsychotics such as olanzapine, however, controlled studies have not demonstrated efficacy in anorexia nervosa patients.^51^, ^52^, ^53^, ^54^, ^121^ In patients with bulimia nervosa, studies have suggested SSRIs may be beneficial in decreasing the frequency of binge eating and purging.^54^, ^55^, ^56^

#### 4.2.4 Delusional disorder: Delusions of parasitosis

##### Other name: Parasitophobia, delusional infestation, morgellons

Delusions of parasitosis is a delusional psychiatric disorder characterized by the illusion of parasites embedded in the skin which leads to compulsive picking of the skin in attempts to soothe the area or remove the parasite. Patients may present with self induced lesions in areas accessible to the patient and may arrive with a scraping of skin which they believe to contain the parasite.^6^, ^57^ Delusions of parasitosis is classified as a delusional disorder in the DSM‐5.^32^

Primary delusional parasitosis is when the patient has a delusion of being infested but has no other organic or psychiatric disorders that are present. Secondary and organic forms can occur secondary to other conditions such as psychiatric or and organic disease. Delusions of parasitosis is a diagnosis of exclusion, therefore, the dermatologist must first evaluate for the presence of parasites. If none are identified, medical causes should be ruled out or screened for such as hypothyroidism, vitamin B12 deficiency, neuropathy, diabetes mellitus, HIV, syphilis, tuberculosis, leprosy, other psychiatric disorders (schizophrenia), neurological causes such as multiple sclerosis or a stroke, alcohol withdrawal, cocaine abuse, amphetamine abuse, and adverse effect of medication. Once delusions of parasitosis are diagnosed, general measures include emollients and gentle soaps to minimize irritation, wound dressings, and topical antipruritics. The patient should be informed that no parasites were found on microscopy and educated that the brain may be interpreting sensory information as infestation, however, these symptoms can be treated with medication. The patient should never be misinformed that psychotropic medications can treat the parasite. The first generation antipsychotic pimozide was found to be efficacious, but the second‐generation antipsychotics risperidone, olanzapine, quetiapine, and aripiprazole are now preferable due to more favourable side effect profiles.^57^, ^58^, ^123^

Common psychiatric comorbidities include depressive disorders, anxiety disorders, OCD, personality disorders, substance use disorder, BDD, schizophrenia and eating disorders.^6^, ^57^ CBT can be used alone for mild cases and in conjunction with medications for more severe cases.^58^

Often these patients migrate from one provider to another in search of a provider that will agree with them. Many providers are quick to confront the patient and to tell the patient that they are wrong and then the patient then moves on causing a staggering burden to the cost of healthcare.^124^ One example in working with these patients is to initiate aspects of CBT where the physician can act as their ally to build trust and point out in a logical manner their abnormal psychosocial behaviour. The physician should take the bag of ‘insects’ or specimens that the patient claims is ‘full of insects’ and look at them with a magnifier or microscope and then indicate that they do not see what they are seeing. However, relate to the patient that you will send the specimen for independent analysis. Then see the patient back in person and show them the lab report which will clearly indicate no evidence of parasites. This should help the patient begin to question their own beliefs. This will also help dispel the shared delusion that the patient's significant other could be harbouring to cope with the psychosocial behaviour. Once some form of trust has been established a multi‐disciplinary approach using mental health experts could be initiated.^57^

### 4.3 Psychogenic excoriations

#### 4.3.1 Acne excoriée

Acne excoriée is a psychiatric involvement of acne vulgaris characterized by excoriated acne eruptions. Patients are self conscious of the eruptions and tend to squeeze or pick lesions repetitively or compulsively in hopes to clear the lesions.^50^, ^51^, ^52^, ^53^, ^54^, ^55^, ^56^, ^57^, ^58^, ^59^ As is the case in most excoriation disorders, picking of the lesions reduces tension which reinforces the behaviour.^60^ Acne excoriée is classified as an obsessive compulsive related disorder in the DSM‐5.^32^

Treatment of the underlying acne vulgaris is outlined above under the heading ‘acne vulgaris’. Careful cleaning and proper hygiene can help with avoidance of face picking.^60^ The excoriation behaviour of acne excoriée is not correlated with the severity of the disease. Repetitive manipulation of the lesions can lead to scarring, hyperpigmentation, and even mutilation, so early psychiatric intervention is preferred.^60^

The most common psychiatric comorbidities are anxiety disorders, depressive disorders, substance use disorder, and BDD.^61^, ^62^ Behavioural therapies are especially helpful and include CBT, habit reversal therapy, and acceptance‐enhanced behavioural therapy. Various psychotropic medications can be used and include SSRIs, the anxiolytics lorazepam and buspirone, the antipsychotics olanzapine and paliperidone, naltrexone, and N‐acetylcysteine.^59^, ^60^

Much like the example in lichen simplex chronicus, the use of masking tape with written reminders can be helpful in increasing mindfulness and reinforcing that the behaviour is destructive and contributes to the condition.^59^, ^60^

#### 4.3.2 Dermatillomania

##### Other name: Skin picking disorder

Dermatillomania is characterized by recurrent compulsive picking of the skin which may lead to severe damage to the skin and physical disfigurement. Self induced lesions may be observed on the face, legs, arms, torso, hands, cuticles, fingers, and scalp, with the face being the most common area affected. Recurrence of the behaviour is reinforced by the relief of tension and for some patients, a pleasurable sensation.^6^, ^61^ The pathophysiology is thought to involve abnormalities in neurotransmitter metabolism, specifically involving serotonin, dopamine, and glutamate.^6^ Dermatillomania is classified as an obsessive compulsive‐related disorder in the DSM‐5.^32^

The dermatologist should first address and manage the lesions while simultaneously fostering a relationship with the patient. Wound cleaning, emollients, and other topical therapies may be used to manage wounds and reinforce trust. Antidepressants, especially SSRIs, may be employed to aid in control and compulsion of symptoms.^41^

Common psychiatric comorbidities include anxiety disorders, depressive disorders, OCD, BDD, substance use disorder, dissociative disorders, obsessive‐compulsive personality disorder, and BPD.^6^, ^41^ Nonpharmacologic treatment modalities include psychotherapy, especially CBT, psychodynamic, or habit reversal therapy; transcranial magnetic stimulation, and supplemental therapies such as psychoeducation, yoga, meditation, mindfulness, aerobic exercise, relaxation techniques, hypnosis, acupuncture, and biofeedback.^9^, ^41^, ^59^ Psychotropic medications that may be prescribed include SSRIs, with fluoxetine showing the most success; venlafaxine, atypical antipsychotics, anxiolytics, naltrexone, lamotrigine, and glutamatergic agents such as N‐acetylcysteine.^6^, ^41^, ^59^

#### 4.3.3 Onychotillomania

##### Other name: Onychophagia

Onychotillomania is a psychiatric disorder characterized by recurrent and compulsive fingernail biting or picking, closely related to stress. Affected areas may include fingernails, cuticles, or bordering skin.^63^, ^64^, ^125^ Onychotillomania is classified as an obsessive compulsive related disorder in the DSM‐5.^32^

Treatment depends on the severity of the disorder. Fungal or bacterial infections may develop around the nail bed and require treatment with antimicrobials. The dermatologist may recommend keeping the nails short, manicured, or covered. A bitter nail polish may be painted on the nails to discourage biting. Behaviour modifications are recommended such as occupying hands with a stress ball and identifying or managing triggers.^126^ Complications of this disorder include nail bed damage, stomach and intestinal infections, temporomandibular joint pain and dysfunction, root resorption of teeth, malocclusion, gingiva injuries, and alveolar destruction.^63^, ^64^, ^65^, ^125^

The most common psychiatric comorbidities include depressive disorders, OCD, attention deficit/hyperactivity disorders, oppositional defiant disorder, separation anxiety disorder, enuresis, and tic disorders.^63^, ^64^ Psychotherapy such as CBT or habit reversal therapy may be helpful in remitting the behaviour.^63^ The medications fluoxetine, clomipramine, and desipramine have been successfully used.^63^ SSRIs are the treatment of choice in more severe cases.^64^

#### 4.3.4 Trichotillomania

##### Other name: Hair pulling disorder

Trichotillomania is a psychiatric disorder characterized by recurrent hair pulling which leads to variable hair loss which may or may not be noticeable to others. Affected areas may include the scalp, eyebrows, eyelashes, beard, trunk, armpits, and pubic area, with scalp being the most common. In affected areas, short, broken strands of hair may be observed with long, normal hairs or overt bald patches may be present. Evidence of other acts of self mutilation may also be seen. It is theorized that the pathophysiology involves abnormalities with the basal ganglia and the neurotransmitters serotonin, dopamine, norepinephrine, and glutamate. It may also develop as an early coping mechanism in response to stress that is reinforced by tension relief.^6^, ^40^, ^64^, ^65^ Trichotillomania is classified as an obsessive compulsive related disorder in the DSM‐5.^32^

The dermatologist may suggest gloves or socks to cover the hands or cutting the hair close to the scalp. Topical steroids, hydroxyzine, N‐acetylcysteine, SSRIs, and TCAs are medications that have shown efficacy. Topical minoxidil may be recommended to potentiate hair regrowth.^40^, ^64^, ^65^

This disorder has various documented psychiatric comorbidities including OCD, depressive disorders, anxiety disorders, Tourette's disorder, eating disorders, substance use disorder, and personality disorders: obsessive compulsive, borderline, and narcissistic. Behavioural treatments such as biofeedback, self‐monitoring, desensitization, habit reversal, insight oriented psychotherapy, dialectical behaviour therapy, CBT, and hypnotherapy are useful. Medications that show efficacy for trichotillomania include SSRIs, especially fluvoxamine, sertraline, and citalopram; anxiolytics, the antipsychotics olanzapine and aripiprazole; venlafaxine, clomipramine, naltrexone, oxcarbazepine, lithium, buspar, clonazepam, and trazodone. Augmenting a SSRI with pimozide may elicit a better response.^6^, ^40^, ^41^, ^59^, ^65^

A helpful example for the manias (dermatillomania, onychotillomania, trichotillomania) would be to use sublimation therapy. One could teach the patient to do other actions such as squeezing stress balls, hitting a portable punching bag or any other physical or mental activity that will cease the noxious behaviour. The goal would be to stop the self harm and focus these energies elsewhere away from the body. Many other sublimation therapies could be art therapy and/or taking up hobbies that would give the patient pleasurable feedback.^66^

## 5 DISCUSSION

There are numerous psychotherapeutic interventions at the dermatologist's disposal that one should be aware of in order to provide the highest quality of care (Tables 1 and 2). Figure 1 graphically shows a concentration of primary dermatologic conditions associated with 3 major DSM‐5 classifications: depressive disorders, anxiety disorders, and obsessive compulsive disorders. This information that was gleaned from the review of the literature helps both the dermatologist and the mental health professional understand what core issues must be addressed for resolution of the presenting problem. When treating multiple dermatologic and or psychiatric disorders, it is crucial for healthcare providers to clearly delineate isolated disorders from those which may have underlying roots in a comorbid condition.

**TABLE 2:** Tool box of screening tools and psychotherapeutic interventions^6^, ^8^, ^9^, ^10^, ^11^, ^12^, ^13^, ^14^, ^15^, ^16^, ^17^, ^18^, ^19^, ^20^, ^21^, ^22^, ^23^, ^24^, ^25^, ^26^, ^27^, ^28^, ^29^, ^30^, ^31^, ^32^, ^33^, ^34^, ^35^, ^36^, ^37^, ^38^, ^39^, ^40^, ^41^, ^42^, ^43^, ^127^, ^128^, ^129^, ^130^, ^131^

Psychotherapeutic interventions	Cognitive behaviour therapy (CBT) ‐ CBT is a type of talk therapy that aims to challenge and change distorted thoughts, beliefs, attitudes, and resulting behaviours in order to improve emotional regulation and develop coping strategies Desensitization Exposure and response prevention Acceptance‐enhanced behavioural therapy Mindfulness/meditation Dialectical behaviour therapy (DBT)‐A form of CBT where the therapist treats the patient as an ally. The therapist works together with a patient as their advocate but also points out their abnormal psychosocial behaviour and makes recommendations on improving this behaviour. This form of therapy uses multiple tools to achieve these goals including having the patient do a diary, practice meditation and other supplemental therapies to achieve the goal of emotional balance
Supplemental therapies	Acupuncture Art therapy Biofeedback Hypnosis Journaling/diary Psychoeducation‐insightful information to help patients and family members Yoga
Psycho pharmacological therapy	Antidepressants Selective serotonin reuptake inhibitors (SSRIs)‐ citalopram, dapoxetine escitalopram, fluoxetine fluvoxamine, paroxetine sertraline, voritioxetine Serotonin and norepinephrine reuptake inhibitors (SNRIs) (duloxetine, venlafaxine, milnacipran) Tricyclic antidepressants (TCAs)‐ amitriptyline,amoxapine, desipramine, doxepin, imipramine, nortriptyline, protriptyline, trimipramime Monamine oxidase inhibitors (MAOIs)‐ selegiline, isoarboxazid, phenelzine, tranylcypromim Anxiolytics‐benzodiazepines‐(Alprazolam,chlordiazepoxide, clonazepam, clorazepate, diazepam, estazolam, flurazepam, lorazepam) Anxiolytics (buspirone) Opioid antagonist‐naltrexone Antipsychotics (pimozide, olanzapine, risperidone, aripiprazole, paliperidone) Anticonvulsants (oxcarbazepine) Other: lithium,N‐acetylcysteine, lamotrigine
Screening tools	Patient health questionnaire 2 and 9 (PHQ‐2)(PHQ‐9) to screen for depression General anxiety disorder 7 (GAD‐7) questionnaire to screen for anxiety Yale‐Brown obsessive compulsive scale (Y‐BOCS) to screen for obsessive compulsive disorders Sick, control, one, fat and food (SCOFF) to screen for eating disorders Body dysmorphic disorder questionnaire (BDDQ) to screen for body dysmorphic disorder

There are surprising comorbidities that can be examined in Figure 1. In those with psoriasis, personality disorders like schizoid and narcissistic personality disorder may go unnoticed by many clinicians. Investigating comorbidities like these have the potential to quiesce the distress and loss of productivity due to the psychiatric condition, while simultaneously blunting flares of the dermatologic condition. For example, treatment of BPD with dialectical behavioural therapy could theoretically improve the outcomes of the personality disorder and the prognosis for a patient suffering from severe psoriasis.^132^ Further investigation into the diminution of dermatologic symptoms using primarily, or solely, psychiatric modalities seems to be a promising area of research exploration. Since the overlap of psychiatric and dermatologic disease is significant, it could be beneficial to incorporate into the established dermatologic clinical assessment the use of validated screening tools such as the patient health questionnaire 2 and 9 (PHQ‐2) (PHQ‐9) to screen for depression, general anxiety disorder 7 questionnaire to screen for anxiety, and the Yale‐Brown obsessive compulsive scale to screen for obsessive compulsive disorders.^127^, ^128^, ^129^ The sick, control, one, fat and food (SCOFF) questionnaire for eating disorders and the BDD questionnaire (BDDQ) may also be incorporated to screen for other psychiatric comorbidities.^130^, ^131^ The PHQ‐2 consists of two questions asking if depressed mood or anhedonia have been experienced over the past 2 weeks. If they respond ‘yes’ to either question, they should be given the PHQ‐9. The PHQ‐9 contains those first two questions followed by 7 more and each question is scored based on how often the patient experiences the symptoms. The final score classifies the severity of the depression using defined categories of mild, moderate, moderately severe, and severe.^131^ Moderate scores of 10 and higher indicate that psychotherapy or psychotropic medication should be considered.^133^ This holds implications for the dermatologist as the dermatologist could use the PHQ‐9 to make the call to refer the patient to a psychiatrist or other mental health professional, or start the patient on a psychotropic medication and monitor the patient, if they feel comfortable doing such. The length of time needed to complete the PHQ‐9 may make this impractical for the dermatologist and therefore the PHQ‐2 may be a more practical tool. If a patient screens positive on the PHQ‐2, they can be referred to a psychiatrist or therapist to complete the more in depth PHQ‐9.^133^ The anxiety and depression association of America has self‐screening questions for multiple mental health disorders including but not limited to: depressive disorders, anxiety disorders, and OCD. Dermatologists would instruct patients to complete the self‐screening, print out their responses or upload into a patient portal. These questionnaires provide the dermatologist with easily interpretable data that can aid in proper diagnosis and course of treatment of dermatologic conditions with underlying psychiatric comorbidities. At this point, the patient would be referred to a mental health practitioner for a more in‐depth work‐up and psychiatric treatment. We believe this approach will result in greater alleviation of symptoms as well as better long‐term treatment outcomes. For example, pruritus intensity has been found to correlate with the severity of depression.^134^ Treating underlying depression and other psychiatric disorders may streamline treatment and achieve better control of symptoms such as pruritus. This also holds implications for future research as clinicians could consider constructing and trialing a specific questionnaire that would screen for multiple mental health disorders as they relate to dermatologic conditions. It may be instructive to request patients fill out these questionnaires as part of their periodic routine paperwork, after initial rapport has been established.

Regarding the primary psychiatric disorders with dermatologic manifestations, patients will typically see specialists other than the psychiatrist, such as the dermatologist, to address their medical concerns.^6^ Since the patient may be reluctant or flat out refuse to see a psychiatrist, the dermatologist can use Table 2 as well as the information above to guide potential treatment modalities. Of note, our aim is not to encourage dermatologists to prescribe psychotropic medications or psychotherapeutic modalities without specific training, but to provide information for better management of psychocutaneous disorders in collaboration with mental health professionals. For patients highly resistant to a psychiatric evaluation, these authors believe it would be beneficial if there was a mechanism in place whereby a dermatologist, or any health care provider, could consult with a psychiatrist to aid in implementation of therapy with the aim of transfer of care to that psychiatrist.

## 6 CONCLUSION

It is clear from the review of the literature that more work needs to be done on understanding the association between dermatologic and psychiatric disorders. It is also clear that the associations found narrows our focus for better therapeutics, whether they be dermatologic or psychiatric. It would be important to further study the hyper concentration of the 3 DSM‐5 classifications of depressive disorders, anxiety disorders, and obsessive compulsive disorders, and possibly come up with a universal screening tool that would incorporate these three major classifications. It is also important to understand the mechanism for psychiatric outliers and how to better address the needs of the patient and the efficiency of the clinician through streamlining therapies.

## CONFLICT OF INTEREST

None to declare.

## AUTHOR CONTRIBUTIONS

**Mary Zagami**: Conceptualization (Lead), Data curation (Lead), Investigation (Lead), Methodology (Lead), Visualization (Equal), Writing – original draft (Lead), Writing – review & editing (Supporting). **Edward Klepper**: Conceptualization (Equal), Data curation (Supporting), Methodology (Equal), Project administration (Equal). Software (Lead), Supervision (Equal), Visualization (Lead), Writing – original draft (Equal), Writing – review & editing (Lead). **Eric Wienecke**: Conceptualization (Equal), Data curation (Supporting), Methodology (Equal), Writing – original draft (Equal), Writing – review & editing (Supporting). **Maria Andrzejewski**: Conceptualization (Equal), Project administration (Equal), Supervision (Equal), Writing – original draft (Equal), Writing – review & editing (Equal). **Ahmed Sikder**: Conceptualization (Supporting), Writing – original draft (Supporting), Writing – review & editing (Supporting). **Ali Ahmed**: Conceptualization (Supporting), Writing – original draft (Supporting), Writing – review & editing (Supporting). **Howard Robinson**: Conceptualization (Equal), Project administration (Lead), Supervision (Lead), Writing – original draft (Supporting), Writing – review & editing (Equal).

## ETHICS STATEMENT

Not applicable.

## Data Availability

Data sharing not applicable, no new data generated.

## References

[1] Jafferany M . Psychodermatology: a guide to understanding common psychocutaneous disorders. Prim Care Companion J Clin Psychiatry. 2007;9:203–13. [PMID: 17632653]. 17632653 10.4088/pcc.v09n0306 PMC1911167

[2] Gieler U , Consoli SG , Tomás‐Aragones L , Linder D , Jemec G , Poot F , et al. Self‐inflicted lesions in dermatology: terminology and classification—a position paper from the European Society for Dermatology and Psychiatry (ESDaP). Acta Derm Venereol. 2013;93(1):4–12. [PMID: 23303467]. 10.2340/00015555-1506 23303467

[3] Lee CS , Accordino R , Howard J , Koo J . Psychopharmacology in dermatology. Dermatol Ther. 2008;21(1):69–82. [PMID: 18318888]. 10.1111/j.1529-8019.2008.00172.x 18318888

[4] Silverberg JI , Silverberg NB . Epidemiology and extracutaneous comorbidities of severe acne in adolescence: a U.S. population‐based study. Br J Dermatol. 2014;170(5):1136–42. [PMID:24641612]. 10.1111/bjd.12912 24641612

[5] Kaymak Y , Taner E , Taner Y . Comparison of depression, anxiety and life quality in acne vulgaris patients who were treated with either isotretinoin or topical agents. Int J Dermatol. 2009;48(1):41–6. [PMID: 19126049]. 10.1111/j.1365-4632.2009.03806.x 19126049

[6] Sadock BJ , Sadock VA , Ruiz P . Kaplan & Sadock's synopsis of psychiatry. 11th ed. Philadelphia: Wolters Kluwer; 2015. p. 424–6.

[7] Zhao ZQ , Liu XY , Jeffry J , Karunarathne W , Li JL , Munanairi A , et al. Descending control of itch transmission by the serotonergic system via 5‐HT1A‐facilitated GRP‐GRPR signaling. Neuron. 2014;84(4):821–34. [PMID: 25453842]. 10.1016/j.neuron.2014.10.003 25453842 PMC4254557

[8] Singam V , Rastogi S , Patel KR , Lee HH , Silverberg JI . The mental health burden in acne vulgaris and rosacea: an analysis of the US National Inpatient Sample. Clin Exp Dermatol. 2019;44(7):766–72. [PMID: 30706514]. 10.1111/ced.13919 30706514

[9] Tusaie KR , Fitzpatrick JJ . Advanced practice psychiatric nursing: integrating psychotherapy, psychopharmacology, and complementary and alternative approaches across the life span. New York: Springer Publishing Company, LLC; 2017.

[10] Harries M , Macbeth AE , Holmes S , Thompson AR , Chiu WS , Gallardo WR , et al. Epidemiology, management and the associated burden of mental health illness, atopic and autoimmune conditions, and common infections in alopecia areata: protocol for an observational study series. BMJ Open. 2021;11:e045718. [PMID: 34785540]. 10.1136/bmjopen-2020-045718 PMC8596050 34785540

[11] Marahatta S , Agrawal S , Adhikari BR . Psychological impact of alopecia areata. Dermatol Res Pract. 2020;2020:8879343–5. [PMID: 33424962]. 10.1155/2020/8879343 33424962 PMC7775172

[12] Senra MS , Wollenberg A . Psychodermatological aspects of atopic dermatitis. Br J Dermatol. 2014;170:38–43. [PMID: 24930567]. 10.1111/bjd.13084 24930567

[13] Le Bris V , Chastaing M , Schollhammer M , Brenaut E , Misery L . Usefulness of psychiatric intervention in a joint consultation for the treatment of burning mouth syndrome: a monocentric retrospective study. Acta Derm Venereol. 2019;99(9):813–17. [PMID: 30460375]. 10.2340/00015555-3094 30460375

[14] Bloch RM , Meggs WJ . Comorbidity patterns of self‐reported chemical sensitivity, allergy, and other medical illnesses with anxiety and depression. J Nutr Environ Med. 2007;16(2):136–48. 10.1080/13590840701352823

[15] Ebrahimi A , Rezaei M , Khazaei H , Kavoussi H , Nani N . The association between irritant contact dermatitis of hand and obsessive compulsive disorder in women: a case control study. Dermatol Cosmet. 2013;4:61–8.

[16] Altunay İK , Özkur E , Uğurer E , Baltan E , Aydin C , Serin E . More than a skin disease: stress, depression, anxiety levels, and serum neurotrophins in lichen simplex chronicus. An Bras Dermatol. 2021;96(6):700–5. [PMID: 34620525]. 10.1016/j.abd.2021.04.011 34620525 PMC8790192

[17] Singam V , Patel KR , Silverberg JI . Association of prurigo nodularis and lichen simplex chronicus with hospitalization for mental health disorders in US adults. Arch Dermatol Res. 2020;312(8):587–93. [PMID: 32078024]. 10.1007/s00403-020-02046-5 32078024

[18] Yalçın M , Baş A , Ergelen M , Gokce E , Usta Saglam NG , Ocek Bas T , et al. Psychiatric comorbidity and temperament‐character traits of the patients with lichen simplex chronicus: the relation with the symptom severity of the disease. Dermatol Ther. 2020;33(6):e14389. [PMID: 33034929]. 10.1111/dth.14389 33034929

[19] Schneider G , Pogatzki‐Zahn E , Marziniak M , Stumpf A , Ständer S . Cutaneous sensory function is not related to depression and anxiety in patients with chronic pruritus with dysesthetic subqualities. Acta Derm Venereol. 2015;95(3):289–93. [PMID: 25111503]. 10.2340/00015555-1933 25111503

[20] Fabrazzo M , Cipolla S , Signoriello S , Camerlengo A , Calabrese G , Giordano GM , et al. A systematic review on shared biological mechanisms of depression and anxiety in comorbidity with psoriasis, atopic dermatitis, and hidradenitis suppurativa. Eur Psychiatr. 2021;64(1):e71. [PMID: 34819201]. 10.1192/j.eurpsy.2021.2249 PMC8668448 34819201

[21] Pompili M , Innamorati M , Forte A , Erbuto D , Lamis DA , Narcisi A , et al. Psychiatric comorbidity and suicidal ideation in psoriasis, melanoma and allergic disorders. Int J Psychiatr Clin Pract. 2017;21(3):209–14. [PMID: 28326880]. 10.1080/13651501.2017.1301482 28326880

[22] Remröd C , Sjöström K , Svensson Å . Pruritus in psoriasis: a study of personality traits, depression and anxiety. Acta Derm Venereol. 2015;95(4):439–43. [PMID: 25229695]. 10.2340/00015555-1975 25229695

[23] Jafferany M , Bhattacharya G . Psychogenic purpura (Gardner‐Diamond syndrome). Prim Care Companion CNS Disord. 2015;17. [PMID: 26137346]. 10.4088/PCC.14br01697 PMC4468873 26137346

[24] Nazik H , Mülayim MK , Öztürk P , Kazancı U , Sarı ID . Report of two cases with autoerythrocyte sensitization syndrome. Ķazaķstannyṇ Klinikalyķ Medicinasy. 2018;4(50):40–3. 10.23950/1812-2892-JCMK-00632

[25] Rainer BM , Kang S , Chien AL . Rosacea: epidemiology, pathogenesis, and treatment. Dermatoendocrinol. 2017;9(1):e1361574. [PMID: 29484096]. 10.1080/19381980.2017.1361574 29484096 PMC5821167

[26] Cömert A , Akbaş B , Kılıç EZ , Akin O , Gokce E , Goktuna Z , et al. Psychiatric comorbidities and alexithymia in patients with seborrheic dermatitis: a questionnaire study in Turkey. Am J Clin Dermatol. 2013;14(4):335–42. [PMID: 23609607]. 10.1007/s40257-013-0019-7 23609607

[27] Orion E , Wolf R . Psychologic factors in the development of facial dermatoses. Clin Dermatol. 2014;32(6):763–6. [PMID: 25441469]. 10.1016/j.clindermatol.2014.02.015 25441469

[28] Jerković H , Bešlić I , Ćesić D , Šitum M . The psychosocial burden of urticaria. Rad Hrvat Akad Znan i Umjet Med Znan. 2021;548:98–103. 10.21857/9xn31cogdy

[29] Ramakrishna P , Rajni T . Psychiatric morbidity and quality of life in vitiligo patients. Indian J Psychol Med. 2014;36(3):302–3. [PMID: 25035556]. 10.4103/0253-7176.135385 25035556 PMC4100418

[30] Sadownik LA . Etiology, diagnosis, and clinical management of vulvodynia. Int J Womens Health. 2014;6:437–49. [PMID: 24833921]. 10.2147/ijwh.s37660 24833921 PMC4014358

[31] Bergeron S , Likes WM , Steben M . Psychosexual aspects of vulvovaginal pain. Best Pract Res Clin Obstet Gynaecol. 2014;28(7):991–9. [PMID: 25104563]. 10.1016/j.bpobgyn.2014.07.007 25104563

[32] Diagnostic and statistical manual of mental disorders: DSM‐5. 5th ed. Arlington: American Psychiatric Association, 2013.

[33] Phillips KA , Hollander E . Treating body dysmorphic disorder with medication: evidence, misconceptions, and a suggested approach. Body Image. 2008;5(1):13–27. [PMID: 18325859]. 10.1016/j.bodyim.2007.12.003 18325859 PMC2705931

[34] Hong K , Nezgovorova V , Uzunova G , Schlussel D , Hollander E . Pharmacological treatment of body dysmorphic disorder. Curr Neuropharmacol. 2019;17(8):697–702. [PMID: 29701157]. 10.2174/1570159x16666180426153940 29701157 PMC7059151

[35] Gunstad J , Phillips KA . Axis I comorbidity in body dysmorphic disorder. Compr Psychiatr. 2003;44(4):270–6. [PMID: 12923704]. 10.1016/s0010-440x(03)00088-9 PMC1613797 12923704

[36] Rautio D , Jassi A , Krebs G , Andren P , Monzani B , Gumpert M , et al. Clinical characteristics of 172 children and adolescents with body dysmorphic disorder. Eur Child Adolesc Psychiatr. 2022;31(1):133–44. [PMID: 33165651]. 10.1007/s00787-020-01677-3 PMC8817062 33165651

[37] Harrison A , Fernández de la Cruz L , Enander J , Radua J , Mataix‐Cols D . Cognitive‐behavioral therapy for body dysmorphic disorder: a systematic review and meta‐analysis of randomized controlled trials. Clin Psychol Rev. 2016;48:43–51. [PMID: 27393916]. 10.1016/j.cpr.2016.05.007 27393916

[38] Hariharasubramony A , Chankramath S , Srinivasa S . Munchausen syndrome as dermatitis simulata. Indian J Psychol Med. 2012;34(1):94–6. [PMID: 22661819]. 10.4103/0253-7176.96171 22661819 PMC3361855

[39] Lavery MJ , Stull C , McCaw I , Anolik RB . Dermatitis artefacta. Clin Dermatol. 2018;36(6):719–22. [PMID: 30446194]. 10.1016/j.clindermatol.2018.08.003 30446194

[40] Wong JW , Nguyen TV , Koo JY . Primary psychiatric conditions: dermatitis artefacta, trichotillomania and neurotic excoriations. Indian J Dermatol. 2013;58(1):44–8. [PMID: 23372212]. 10.4103/0019-5154.105287 23372212 PMC3555372

[41] Tomas‐Aragones L , Consoli SM , Consoli SG , Poot F , Taube K , Linder M , et al. Self‐inflicted lesions in dermatology: a management and therapeutic approach – a position paper from the European society for dermatology and psychiatry. Acta Derm Venereol. 2017;97(2):159–72. [PMID: 27563702]. 10.2340/00015555-2522 27563702

[42] Pradhan S , Sirka CS , Dash G , Mohapatra D . Dermatitis artefacta in a child: an interesting morphological presentation. Indian Dermatol Online J. 2019;10(1):72. [PMID: 30775305]. 10.4103/idoj.idoj_132_18 30775305 PMC6362749

[43] Hay PJ , Claudino AM . Bulimia nervosa. BMJ Clin Evid. 2010;2010(7303):1009–37. [PMID: 21418667]. 10.1136/bmj.323.7303.33 PMC3275326 21418667

[44] Yager J , Andersen AE . Clinical practice. Anorexia nervosa. N Engl J Med. 2005;353(14):1481–8. [PMID: 16207850]. 10.1056/nejmcp050187 16207850

[45] Carter FA , Jordan J , McIntosh VV , Luty SE , McKenzie JM , Frampton CM , et al. The long‐term efficacy of three psychotherapies for anorexia nervosa: a randomized, controlled trial. Int J Eat Disord. 2011;44(7):647–54. [PMID: 21997429]. 10.1002/eat.20879 21997429

[46] Murphy R , Straebler S , Cooper Z , Fairburn CG . Cognitive behavioral therapy for eating disorders. Psychiatr Clin. 2010;33(3):611–27. [PMID: 20599136]. 10.1016/j.psc.2010.04.004 PMC2928448 20599136

[47] Selby A , Smith‐Osborne A . A systematic review of effectiveness of complementary and adjunct therapies and interventions involving equines. Health Psychol. 2013;32(4):418–32. [PMID: 22888815]. 10.1037/a0029188 22888815

[48] Klein J , Cook‐Cottone C . The effects of yoga on eating disorder symptoms and correlates: a review. Int J Yoga Therap. 2013;23(2):41–50. [PMID: 24165522]. 10.17761/ijyt.23.2.2718421234k31854 24165522

[49] Attia E , Haiman C , Walsh BT , Flater SR . Does fluoxetine augment the inpatient treatment of anorexia nervosa? Am J Psychiatr. 1998;155(4):548–51. [PMID: 9546003]. 10.1176/ajp.155.4.548 9546003

[50] Walsh BT , Kaplan AS , Attia E , Olmsted M , Parides M , Carter JC , et al. Fluoxetine after weight restoration in anorexia nervosa: a randomized controlled trial. JAMA. 2006;295(22):2605–12. [PMID: 16772623]. 10.1001/jama.295.22.2605 16772623

[51] McKnight RF , Park RJ . Atypical antipsychotics and anorexia nervosa: a review. Eur Eat Disord Rev. 2010;18(1):10–21. [PMID: 20054875]. 10.1002/erv.988 20054875

[52] Bissada H , Tasca GA , Barber AM , Bradwejn J . Olanzapine in the treatment of low body weight and obsessive thinking in women with anorexia nervosa: a randomized, double‐blind, placebo‐controlled trial. Am J Psychiatr. 2008;165(10):1281–8. [PMID: 18558642]. 10.1176/appi.ajp.2008.07121900 18558642

[53] Kafantaris V , Leigh E , Hertz S , Berest A , Schebendach J , Sterling WM , et al. A placebo‐controlled pilot study of adjunctive olanzapine for adolescents with anorexia nervosa. J Child Adolesc Psychopharmacol. 2011;21(3):207–12. [PMID: 21663423]. 10.1089/cap.2010.0139 21663423

[54] Hay PJ , Claudino AM . Clinical psychopharmacology of eating disorders: a research update. Int J Neuropsychopharmacol. 2012;15(02):209–22. [PMID: 21439105]. 10.1017/s1461145711000460 21439105

[55] Capasso A , Petrella C , Milano W . Pharmacological profile of SSRIs and SNRIs in the treatment of eating disorders. Curr Clin Pharmacol. 2009;4(1):78–83. [PMID: 19149506]. 10.2174/157488409787236092 19149506

[56] Milano W , Petrella C , Sabatino C , Capasso A . Treatment of bulimia nervosa with sertraline: a randomized controlled trial. Adv Ther. 2004;21(4):232–7. [PMID: 15605617]. 10.1007/bf02850155 15605617

[57] Reich A , Kwiatkowska D , Pacan P . Delusions of parasitosis: an update. Dermatol Ther. 2019;9(4):631–8. [PMID: 31520344]. 10.1007/s13555-019-00324-3 PMC6828902 31520344

[58] Lepping P , Russell I , Freudenmann RW . Antipsychotic treatment of primary delusional parasitosis: systematic review. Br J Psychiatry. 2007;191(3):198–205. [PMID: 17766758]. 10.1192/bjp.bp.106.029660 17766758

[59] Lochner C , Roos A , Stein DJ . Excoriation (skin‐picking) disorder: a systematic review of treatment options. Neuropsychiatric Dis Treat. 2017;13:1867–72. [PMID: 28761349]. 10.2147/ndt.s121138 PMC5522672 28761349

[60] Anzengruber F , Ruhwinkel K , Ghosh A , Klaghofer R , Lang UE , Navarini AA . Wide range of age of onset and low referral rates to psychiatry in a large cohort of acne excoriée at a Swiss tertiary hospital. J Dermatol Treat. 2018;29(3):277–80. [PMID: 28784003]. 10.1080/09546634.2017.1364693 28784003

[61] Gieler U , Gieler T , Peters EMJ , Linder D . Skin and psychosomatics – psychodermatology today. J Dtsch Dermatol Ges. 2020;18(11):1280–98. [PMID: 33251751]. 10.1111/ddg.14328 PMC7756276 33251751

[62] Kwon C , Sutaria N , Khanna R , Almazan E , Williams K , Kim N , et al. Epidemiology and comorbidities of excoriation disorder: a retrospective case‐control study. J Clin Med. 2020;9:2703. [PMID: 3282562]. 10.3390/jcm9092703 32825621 PMC7564859

[63] Ghanizadeh A . Nail biting; etiology, consequences and management. Iran J Med Sci. 2011;36:73–9. [PMID: 23358880]. 23358880 PMC3556753

[64] Pacan P , Grzesiak M , Reich A , Szepietowski JC . Onychophagia as a spectrum of obsessive‐compulsive disorder. Acta Derm Venereol. 2009;89(3):278–80. [PMID: 19479125]. 10.2340/00015555-0646 19479125

[65] França K , Kumar A , Castillo D , Jafferany M , Hyczy da Costa Neto M , Damevska K , et al. Trichotillomania (hair pulling disorder): clinical characteristics, psychosocial aspects, treatment approaches, and ethical considerations. Dermatol Ther. 2019;32(4):e12622. [PMID: 30152568]. 10.1111/dth.12622 30152568

[66] Snorrason I , Berlin GS , Lee HJ . Optimizing psychological interventions for trichotillomania (hair‐pulling disorder): an update on current empirical status. Psychol Res Behav Manag. 2015;8:105–13. [PMID: 25897268]. 10.2147/prbm.s53977 25897268 PMC4396507

[67] Ash C , Harrison A , Drew S , Whittal R . A randomized controlled study for the treatment of acne vulgaris using high‐intensity 414 nm solid state diode arrays. J Cosmet Laser Ther. 2015;174(4):170–76. [PMID: 25594129]. 10.3109/14764172.2015.1007064 25594129

[68] Zaidi Z . Acne vulgaris‐an update on pathophysiology and treatment. JPMA (J Pak Med Assoc). 2009;59:635–37. [PMID: 19750863]. 19750863

[69] Xu H , Li H . Acne, the skin microbiome, and antibiotic treatment. Am J Clin Dermatol. 2019;20(3):335–44. [PMID: 30632097]. 10.1007/s40257-018-00417-3 30632097 PMC6534434

[70] Chilicka K , Rogowska AM , Szyguła R , Adamczyk E . Association between satisfaction with life and personality types A and D in young women with acne vulgaris. Int J Environ Res Publ Health. 2020;17(22):8524. [PMID: 33212977]. 10.3390/ijerph17228524 PMC7698541 33212977

[71] Yadav S , Narang T , Kumaran MS . Psychodermatology: a comprehensive review. Indian J Dermatol Venereol Leprol. 2013;79(2):176–92. [PMID: 23442456]. 10.4103/0378-6323.107632 23442456

[72] Bosch‐Capblanch X , Abba K , Prictor M , Garner P . Contracts between patients and healthcare practitioners for improving patients' adherence to treatment, prevention and health promotion activities. Cochrane Database Syst Rev. 2007;2007:CD004808. [PMID: 17443556]. 17443556 10.1002/14651858.CD004808.pub3 PMC6464838

[73] Darwin E , Hirt PA , Fertig R , Doliner B , Delcanto G , Jimenez J . Alopecia areata: review of epidemiology, clinical features, pathogenesis, and new treatment options. Int J Trichol. 2018;10(2):51–60. [PMID: 29769777]. 10.4103/ijt.ijt_99_17 PMC5939003 29769777

[74] Gilhar A , Etzioni A , Paus R . Alopecia areata. N Engl J Med. 2012;366(16):1515–25. [PMID: 22512484]. 10.1056/nejmra1103442 22512484

[75] Pratt CH , King LE, Jr , Messenger AG , Christiano AM , Sundberg JP . Alopecia areata. Nat Rev Dis Prim. 2017;3(1):17011. [PMID: 28300084]. 10.1038/nrdp.2017.11 28300084 PMC5573125

[76] Hussain ST , Mostaghimi A , Barr PJ , Brown J , Joyce C , Huang K . Utilization of mental health resources and complementary and alternative therapies for alopecia areata: a U.S. Survey. Int J Trichol. 2017;9(4):160–4. [PMID: 29118520]. 10.4103/ijt.ijt_53_17 PMC5655624 29118520

[77] Gallo R , Chiorri C , Gasparini G , Signori A , Burroni A , Parodi A . Can mindfulness‐based interventions improve the quality of life of patients with moderate/severe alopecia areata? A prospective pilot study. J Am Acad Dermatol. 2017;76(4):757–9. [PMID: 28325394]. 10.1016/j.jaad.2016.10.012 28325394

[78] Boothe D , Tarbox JA , Tarbox MB . Atopic dermatitis: pathophysiology. Adv Exp Med Biol. 2017;1027:21–37. [PMID: 29063428]. 29063428 10.1007/978-3-319-64804-0_3

[79] Boguniewicz M , Leung DY . Atopic dermatitis: a disease of altered skin barrier and immune dysregulation. Immunol Rev. 2011;242(1):233–46. [PMID: 21682749]. 10.1111/j.1600-065x.2011.01027.x 21682749 PMC3122139

[80] Kim K . Neuroimmunological mechanism of pruritus in atopic dermatitis focused on the role of serotonin. Biomol Ther (Seoul). 2012;20(6):506–12. [PMID: 24009842]. 10.4062/biomolther.2012.20.6.506 24009842 PMC3762292

[81] Sanders KM , Akiyama T . The vicious cycle of itch and anxiety. Neurosci Biobehav Rev. 2018;87:17–26. [PMID: 29374516]. 10.1016/j.neubiorev.2018.01.009 29374516 PMC5845794

[82] Schut C , Mollanazar NK , Kupfer J , Gieler U , Yosipovitch G . Psychological interventions in the treatment of chronic itch. Acta Derm Venereol. 2016;96(2):157–6. [PMID: 26073701]. 10.2340/00015555-2177 26073701

[83] Barbarot S , Stalder JF . Therapeutic patient education in atopic eczema. Br J Dermatol. 2014;170(Suppl 1):44–8. [PMID: 24720486]. 10.1111/bjd.12932 24720486

[84] Shi VY , Nanda S , Lee K , Armstrong AW , Lio PA . Improving patient education with an eczema action plan: a randomized controlled trial. JAMA Dermatol. 2013;149(4):481–3. [PMID: 23553035]. 10.1001/jamadermatol.2013.2143 23553035

[85] Nasri‐Heir C , Gomes J , Heir GM , Ananthan S , Benoliel R , Teich S , et al. The role of sensory input of the chorda tympani nerve and the number of fungiform papillae in burning mouth syndrome. Oral Surg Oral Med Oral Pathol Oral Radiol Endod. 2011;112(1):65–72. [PMID: 21601494]. 10.1016/j.tripleo.2011.02.035 21601494

[86] Khan SA , Keaser ML , Meiller TF , Seminowicz DA . Altered structure and function in the hippocampus and medial prefrontal cortex in patients with burning mouth syndrome. Pain. 2014;155(8):1472–80. [PMID: 24769366]. 10.1016/j.pain.2014.04.022 24769366

[87] De Souza IF , Mármora BC , Rados PV , Visioli F . Treatment modalities for burning mouth syndrome: a systematic review. Clin Oral Invest. 2018;22(5):1893–905. [PMID: 29696421]. 10.1007/s00784-018-2454-6 29696421

[88] Lakraj AA , Moghimi N , Jabbari B . Hyperhidrosis: anatomy, pathophysiology and treatment with emphasis on the role of botulinum toxins. Toxins. 2013;5(4):821–40. [PMID: 23612753]. 10.3390/toxins5040821 23612753 PMC3705293

[89] Solish MJ , Savinova I , Weinberg MJ . A practical approach to the diagnosis and treatment of palmar hyperhidrosis. Plast Reconstr Surg Glob Open. 2022;10(3):e4172. [PMID: 35265447]. 10.1097/gox.0000000000004172 35265447 PMC8901220

[90] Schlereth T , Dieterich M , Birklein F . Hyperhidrosis‐causes and treatment of enhanced sweating. Dtsch Arztebl Int. 2009;106:32–7. [PMID: 19564960]. 19564960 10.3238/arztebl.2009.0032 PMC2695293

[91] Stuart ME , Strite SA , Gillard KK . A systematic evidence‐based review of treatments for primary hyperhidrosis. J Drug Assess. 2020;10(1):35–50. [PMID: 33489435]. 10.1080/21556660.2020.1857149 33489435 PMC7781989

[92] Heiskanen SL , Niskala J , Jokelainen J , Tasanen K , Huilaja L , Sinikumpu SP . Hyperhidrosis comorbidities and treatments: a register‐based study among 511 subjects. Acta Derm Venereol. 2022;102:adv00656. [PMID: 35088873]. 10.2340/actadv.v102.1061 35088873 PMC9558338

[93] Klein SZ , Hull M , Gillard KK , Peterson‐Brandt J . Treatment patterns, depression, and anxiety among US patients diagnosed with hyperhidrosis: a retrospective cohort study. Dermatol Ther. 2020;10(6):1299–314. [PMID: 32915394]. 10.1007/s13555-020-00439-y PMC7649188 32915394

[94] Cayir Y , Engin Y . Acupuncture for primary hyperhidrosis: case series. Acupunct Med. 2013;31(3):325–6. [PMID: 23793090]. 10.1136/acupmed-2013-010391 23793090

[95] Welcome MO , Dane S . The effect of acupuncture and foot reflexotherapy on palmar hyperhidrosis in a young girl: a case report. J Res Med Dent Sci. 2020;8:130–5.

[96] Lotti T , Buggiani G , Prignano F . Prurigo nodularis and lichen simplex chronicus. Dermatol Ther. 2008;21(1):42–6. [PMID: 18318884]. 10.1111/j.1529-8019.2008.00168.x 18318884

[97] Torales J , Barrios I , Lezcano L , Di Martino B . Lichen simplex chronicus: easy psychological interventions that every dermatologist should know. SM Dermatolog J. 2016;2(1):1005–5. 10.36876/smdj.1005

[98] Garibyan L , Rheingold CG , Lerner EA . Understanding the pathophysiology of itch. Dermatol Ther. 2013;26(2):84–91. [PMID: 23551365]. 10.1111/dth.12025 23551365 PMC3696473

[99] Song J , Xian D , Yang L , Xiong X , Lai R , Zhong J . Pruritus: progress toward pathogenesis and treatment. BioMed Res Int. 2018;2018:9625936–12. [PMID: 29850592]. 10.1155/2018/9625936 29850592 PMC5925168

[100] Dick MK , Klug MH , Gummadi PP , Klug LK , Huerter CJ . Gardner‐Diamond syndrome: a psychodermatological condition in the setting of immunodeficiency. J Clin Aesthet Dermatol. 2019;12:44–6. [PMID: 32038765]. 32038765 PMC7002047

[101] Akoglu G , Bulut M , Esme P , Akoglu H . Psychogenic purpura (Gardner‐Diamond syndrome) in a hemodialysis patient. Dermatol Ther. 2021;34(2):e14789. [PMID: 33480054]. 10.1111/dth.14789 33480054

[102] Çelik‐Göksoy Ş , Kılınçaslan A , Kaya İ . Psychogenic purpura successfully treated with antidepressant therapy. Antidepresan Tedavi ile İyileşen Psikojenik Purpura. Turk J Haematol. 2017;34:274–5. [PMID: 28270377]. 28270377 10.4274/tjh.2016.0505 PMC5544053

[103] van Zuuren EJ , Fedorowicz Z , Carter B , van der Linden MM , Charland L . Interventions for rosacea. Cochrane Database Syst Rev. 2015;2015:CD003262. [PMID: 25919144]. 25919144 10.1002/14651858.CD003262.pub5 PMC6481562

[104] Borda LJ , Wikramanayake TC . Seborrheic dermatitis and dandruff: a comprehensive review. J Clin Invest Dermatol. 2015;3. [PMID: 27148560]. 10.13188/2373-1044.1000019 PMC4852869 27148560

[105] Janniger CK , Schwartz RA . Seborrheic dermatitis. Am Fam Physician. 1995;52:149–60. [PMID: 7604759]. 7604759

[106] Bracken SJ , Abraham S , MacLeod AS . Autoimmune theories of chronic spontaneous urticaria. Front Immunol. 2019;10:627. [PMID: 30984191]. 10.3389/fimmu.2019.00627 30984191 PMC6450064

[107] Greaves MW . Chronic idiopathic urticaria. Curr Opin Allergy Clin Immunol. 2003;3(5):363–8. [PMID: 14501436]. 10.1097/00130832-200310000-00008 14501436

[108] Grattan C . The urticarias: pathophysiology and management. Clin Med. 2012;12(2):164–7. [PMID: 22586795]. 10.7861/clinmedicine.12-2-164 PMC4954105 22586795

[109] Abdel‐Malek ZA , Jordan C , Ho T , Upadhyay PR , Fleischer A , Hamzavi I . The enigma and challenges of vitiligo pathophysiology and treatment. Pigment Cell Melanoma Res. 2020;33(6):778–87. [PMID: 32198977]. 10.1111/pcmr.12878 32198977

[110] Rashighi M , Harris JE . Vitiligo pathogenesis and emerging treatments. Dermatol Clin. 2017;35(2):257–65. [PMID: 28317534]. 10.1016/j.det.2016.11.014 28317534 PMC5362109

[111] Bornstein J , Goldstein AT , Stockdale CK , Bergeron S , Pukall C , Zolnoun D , et al. 2015 ISSVD, ISSWSH, and IPPS consensus terminology and classification of persistent vulvar pain and vulvodynia. J Sex Med. 2016;13(4):607–12. [PMID: 27008217]. 10.1016/j.jsxm.2016.02.167 27045260

[112] Loflin BJ , Westmoreland K , Williams NT . Vulvodynia: a review of the literature. J Pharm Technol. 2019;35(1):11–24. [PMID: 34861006]. 10.1177/8755122518793256 34861006 PMC6313270

[113] Rosen NO , Dawson SJ , Brooks M , Kellogg‐Spadt S . Treatment of vulvodynia: pharmacological and non‐pharmacological approaches. Drugs. 2019;79(5):483–93. [PMID: 30847806]. 10.1007/s40265-019-01085-1 30847806

[114] Feusner JD , Hembacher E , Moller H , Moody TD . Abnormalities of object visual processing in body dysmorphic disorder. Psychol Med. 2011;41(11):2385–97. [PMID: 21557897]. 10.1017/s0033291711000572 21557897 PMC3913477

[115] Hinney A , Volckmar AL . Genetics of eating disorders. Curr Psychiatr Rep. 2013;15(12):423. [PMID: 24202964]. 10.1007/s11920-013-0423-y 24202964

[116] Treasure J , Zipfel S , Micali N , Wade T , Stice E , Claudino A , et al. Anorexia nervosa. Nat Rev Dis Prim. 2015;26(1):15074. [PMID: 27189821]. 10.1038/nrdp.2015.74 27189821

[117] Watson HJ , Yilmaz Z , Thornton LM , Hübel C , Coleman JRI , Gaspar HA , et al. Genome‐wide association study identifies eight risk loci and implicates metabo‐psychiatric origins for anorexia nervosa. Nat Genet. 2019;51:1207–14. [PMID: 31308545]. 31308545 10.1038/s41588-019-0439-2 PMC6779477

[118] Wonderlich SA , Crosby RD , Mitchell JE , Thompson KM , Redlin J , Demuth G , et al. Eating disturbance and sexual trauma in childhood and adulthood. Int J Eat Disord. 2001;30(4):401–12. [PMID: 11746301]. 10.1002/eat.1101 11746301

[119] Hudson JI , Hiripi E , Pope HG, Jr , Kessler RC . The prevalence and correlates of eating disorders in the National Comorbidity Survey Replication. Biol Psychiatr. 2007;61(3):348–58. [PMID: 16815322]. 10.1016/j.biopsych.2006.03.040 PMC1892232 16815322

[120] Rosen DS , American Academy of Pediatrics Committee on Adolescence . Identification and management of eating disorders in children and adolescents. Pediatrics. 2010;126(6):1240–53. [PMID: 21115584]. 10.1542/peds.2010-2821 21115584

[121] Strumia R . Eating disorders and the skin. Clin Dermatol. 2013;31(1):80–5. [PMID: 23245978]. 10.1016/j.clindermatol.2011.11.011 23245978

[122] Preti A , Rocchi MB , Sisti D , Camboni MV , Miotto P . A comprehensive meta‐analysis of the risk of suicide in eating disorders. Acta Psychiatr Scand. 2011;124(1):6–17. [PMID: 21092024]. 10.1111/j.1600-0447.2010.01641.x 21092024

[123] Davis JM , Chen N , Glick ID . A meta‐analysis of the efficacy of second‐generation antipsychotics. Arch Gen Psychiatr. 2003;60(1):553–64. [PMID: 12796218]. 10.1016/s0920-9964(03)80465-6 12796218

[124] Boggild AK , Nicks BA , Yen L , Van Voorhis W , McMullen R , Buckner FS , et al. Delusional parasitosis: six‐year experience with 23 consecutive cases at an academic medical center. Int J Infect Dis. 2014;14(4):e317–21. [PMID: 19683952]. 10.1016/j.ijid.2009.05.018 19683952

[125] Sachan A , Chaturvedi TP . Onychophagia (Nail biting), anxiety, and malocclusion. Indian J Dent Res. 2012;23(5):680–2. [PMID: 23422619]. 10.4103/0970-9290.107399 23422619

[126] American Academy of Dermatology . Dermatologists share tips to stop nail biting. 2015 [cited 2022 Mar 23]. Available from: www.aad.org/media/news‐releases/dermatologists‐share‐tips‐to‐stop‐nail‐biting

[127] Gilbody S , Richards D , Brealey S , Hewitt C . Screening for depression in medical settings with the Patient Health Questionnaire (PHQ): a diagnostic meta‐analysis. J Gen Intern Med. 2007;22(11):1596–602. [PMID: 17874169]. 10.1007/s11606-007-0333-y 17874169 PMC2219806

[128] Goodman WK , Price LH , Rasmussen SA , Mazure C , Fleischmann RL , Hill CL , et al. The Yale‐Brown Obsessive Compulsive Scale. I. Development, use, and reliability. Arch Gen Psychiatr. 1989;46:1006–11. [PMID: 2684084]. 10.1001/archpsyc.1989.01810110048007 2684084

[129] Spitzer RL , Kroenke K , Williams JB , Löwe B . A brief measure for assessing generalized anxiety disorder: the GAD‐7. Arch Intern Med. 2006;166(10):1092–7. [PMID: 16717171]. 10.1001/archinte.166.10.1092 16717171

[130] Brohede S , Wingren G , Wijma B , Wijma K . Validation of the body dysmorphic disorder Questionnaire in a community sample of Swedish women. Psychiatr Res. 2013;210(2):647–52. [PMID: 23948660]. 10.1016/j.psychres.2013.07.019 23948660

[131] Kutz AM , Marsh AG , Gunderson CG , Maguen S , Masheb RM . Eating disorder screening: a systematic review and meta‐analysis of diagnostic test characteristics of the SCOFF. J Gen Intern Med. 2020;35(3):885–93. [PMID: 31705473]. 10.1007/s11606-019-05478-6 31705473 PMC7080881

[132] Rubino IA , Sonnino A , Pezzarossa B , Ciani N , Bassi R . Personality disorders and psychiatric symptoms in psoriasis. Psychol Rep. 1995;77(2):547–53. [PMID 8559879]. 10.2466/pr0.1995.77.2.547 8559879

[133] Kroenke K , Spitzer RL , Williams JB . The PHQ‐9: validity of a brief depression severity measure. J Gen Intern Med. 2001;16(9):606–13. [PMID: 11556941]. 10.1046/j.1525-1497.2001.016009606.x 11556941 PMC1495268

[134] Schneider G , Driesch G , Heuft G , Evers S , Luger TA , Ständer S . Psychosomatic cofactors and psychiatric comorbidity in patients with chronic itch. Clin Exp Dermatol. 2006;31(6):762–7. [PMID: 17040260]. 10.1111/j.1365-2230.2006.02211.x 17040260

